# Rising serum CA-125 levels within the normal range is strongly associated recurrence risk and survival of ovarian cancer

**DOI:** 10.1186/s13048-020-00681-0

**Published:** 2020-09-02

**Authors:** Szymon Piatek, Grzegorz Panek, Zbigniew Lewandowski, Mariusz Bidzinski, Dominika Piatek, Przemyslaw Kosinski, Miroslaw Wielgos

**Affiliations:** 1 1st Department of Obstetrics and Gynecology, Medical University of Warsaw, 1/3 Starynkiewicza Square, 02-015 Warsaw, Poland; 2 Department of Gynecological Oncology, The Maria Sklodowska-Curie Institute – Oncology Center in Warsaw, 5 Roentgena Street, 02-781 Warsaw, Poland; 3 Department of Epidemiology and Biostatistics, Medical University of Warsaw, 3 Oczki Street, 02-00 Warsaw, Poland; 4 1st Department of Radiology, Medical University of Warsaw, 5 Chałubińskiego Street, 02-004 Warsaw, Poland

**Keywords:** CA-125 changes, CA-125 normal range, Prognostic factor, Overall survival, Progression free survival

## Abstract

**Background:**

In clinical practice alterations in CA-125 concentration within normal range in patients with ovarian cancer after first-line treatment are common. Even minor increase in CA-125 concentration is associated with patients’ anxiety and difficult interpretation and counselling for clinicians. The aim of this study was to evaluate the significance of CA-125 fluctuations within reference level in patients who suffered from ovarian cancer with complete response after first-line treatment.

**Results:**

168 patients with epithelial ovarian cancer, who achieved complete remission after first line treatment were enrolled in the study. CA-125 concentration assessment was carried out during follow up visits. The recurrence of the disease was diagnosed on the first appearance of symptoms: clinical, radiological or histopathological/cytological. PFS and 5-year survival rate was calculated with Kaplan-Meier plots. Statistical analysis was performed with SAS / STAT® 9.4 / 14.4, SAS Institute Inc., Cary, NC, USA, 2017. Median concentration of CA-125 after first-line therapy was 10 U/ml. Increasing CA-125 concentration by > 5 U/ml, 3 and 6 months after the treatment was associated with higher risk of relapse (HR = 7.6, *p* < 0.0001 and HR = 5.29, *p* < 0.0001 respectively). 5-year survival rate was significantly lower in patients with increased CA-125 by 5 U/ml, 3 and 6 months after therapy (56.79% vs 0 and 50.62% vs 15.55%).

**Conclusions:**

Increased concentration of CA-125 by > 5 U/ml within normal range, 3 and 6 months after treatment was unfavorable prognostic factor in ovarian cancer patients with complete response to primary therapy.

## Background

Among gynecological malignancies, ovarian cancer is the leading cause of death in patients in industrialized countries [1]. In Europe, mean-age standardized 5-year relative survival for patients with diagnosed ovarian cancer is 37.6% [2]. This is mainly due to late diagnosis. Only 15% cases are diagnosed as localized diseases [3]. An additional unfavorable factor is the high relapse rate reaching 75%, despite full remission of the disease [4].

According to National Comprehensive Cancer Network recommendations, monitoring of CA-125 concentration is not obligatory for follow-up, but it is common in everyday clinical practice [5]. Any increase in CA-125 concentration arouse anxiety in patients even those in good clinical condition. Patients and clinicians concern lead to redundant referrals and imaging studies. A significant increase in CA-125 concentration (especially those meeting the GCIG recurrence criteria [6]) is unambiguous in interpretation. However, in clinical practice, slight fluctuations of CA-125 concentration are frequently observed. The significance of these changes has not yet been clearly defined. The aim of this study was to assess the significance of changes in CA-125 concentrations within the normal range in patients with complete remission after the first-line treatment.

## Results

**Fig. 1:**
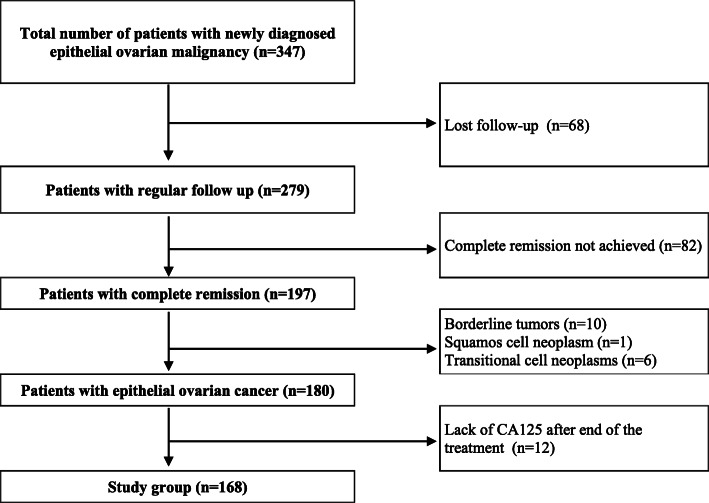
The qualification scheme of patients for the study

This retrospective study included 168 patients treated in the 1st Department of Obstetrics and Gynecology, Medical University of Warsaw and the Department of Gynecologic Oncology, Maria Sklodowska-Curie Oncology Center-Institute in Warsaw from 01/01/2012 to 31/12/2016 (Fig. 1). Detailed characteristics of the study group was presented in Table 1. The study group was predominated patients with advanced ovarian cancer (FIGO III and IV: 69.05%). Most were high grade (G3: 69.6%) tumors and serous histology (73.8%). The median CA-125 concentration at the end of treatment was 10 U/ml. Patients with CA-125 concentration within the normal range 3, 6, 9, 24, 36 months after the end of treatment were 140 (83.3%), 128 (76.2%), 93 (55.3%), 36 (21.4%) and 21 (12.5%).

**Table 1:** Patients’ characteristics

Characteristics		Number (%) / Median (range)	
**Age**			57 (19–86)
**FIGO**			
	I		42 (25%)
	II		10 (5.95%)
	III		103 (61.31%)
	IV		13 (7.74%)
**Histology**			
	Serous		124 (73.81%)
	Endometrioid		18 (10.72%)
	Clear cell		10 (5.95%)
	Mucinous		4 (2.38%)
	Nondifferentiated		4 (2.38%)
	Mixed		8 (4.76%)
**Grade**			
	1		16 (9.52%)
	2		35 (20.83%)
	3		117 (69.65%)
**Cytoreduction**			
	R0		116 (69.05%)
	R1 (≤ 1 cm)		29 (17.26%)
	R2 (> 1 cm)		23 (13.69%)
**Neoadjuvant chemotherapy**			26 (15.48%)
**Bevacizumab**			32 (19.05%)
**CA-125 before therapy**			229.20 U/ml (3.80–6000 U/ml)
**Nadir CA-125 after therapy**			10 U/ml (2.70–35 U/ml)

### CA-125 changes and the risk of relapse

**Fig. 2:**
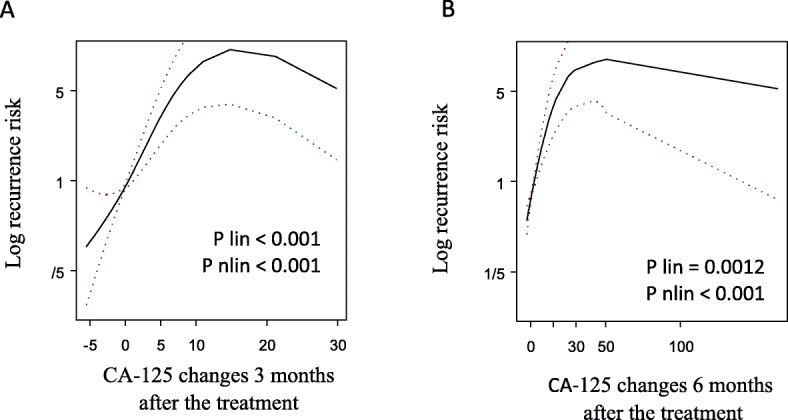
Risk of recurrence according to CA-125 changes 3 (**a**) and 6 (**b**) months after the treatment. Both splins showed statistically significant linear and non-linear increase in recurrence risk 3 and 6 months after the treatment

**Fig. 3:**
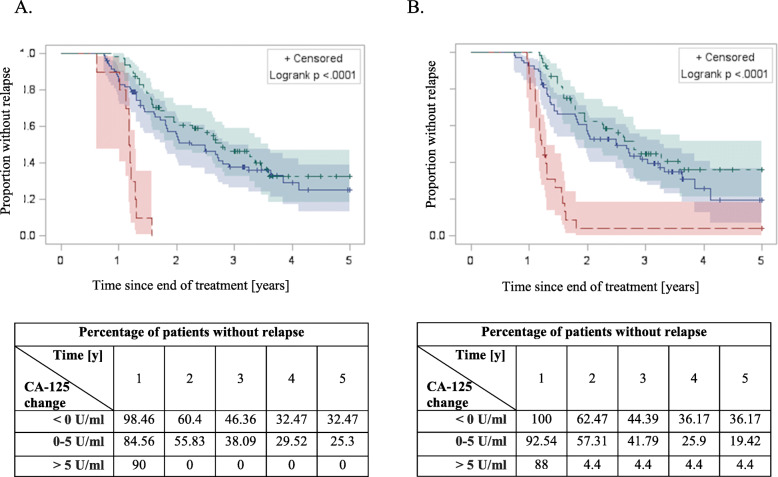
The progression free survival Kaplan-Meier curves among patients with different CA-125 changes 3 (**a**) and 6 (**b**) months after end of the treatment. Green: < 0 U/ml; blue: 0–5 U/ml; red > 5 U/ml. Statistically significant difference in PFS was achieved with log-rank test

A statistically significant correlation was found between the risk of recurrence and the change in CA-125 concentration 3 and 6 months after the end of treatment (Fig. 2a). 3 months after the end of treatment, the risk of recurrence in patients with CA-125 > 5 U/ml was higher than in patients whose CA-125 levels did not increase (HR = 7.6, 95% CI: 3.55–16.24, *p* < 0.0001; Table 2, Fig. 3a). Increase in CA-125 > 5 U/ml 6 months after the end of treatment was also associated with higher risk of recurrence, but the risk was lower (HR = 5.29, 95% CI: 2.99–9.35, *p* < 0.0001; Table 2, Fig. 3b).

**Table 2:** Risk of recurrence and CA-125 changes after the treatment

Variable		HR	95%CI	P
**Change of CA-125 concentration** **3 months after the treatment (U/ml)**	< 0	1.00	–	–
	0–5	1.25	0.80–1.93	0.3287
	> 5	7.60	3.55–16.24	< 0.0001
**Change of CA-125 concentration** **6 months after the treatment (U/ml)**	< 0	1.00	–	–
	0–5	1.31	0.82–2.1	0.2598
	> 5	5.29	2.99–9.35	< 0.0001

### Risk of recurrence/death per 1 U/ml CA-125

In order to compare the risk of recurrence/death between patients, it was calculated per 1 U/ml. It has been shown that an increase in CA-125 concentration by 1 U/ml was associated with higher risk of relapse and death. Any increase in the concentration of CA125 3 and 6 months after end of the treatment by 1 U/ml increased risk of relapse by 8.3 and 1.8% respectively. Risk of death related to CA-125 increase by 1 U/ml 3 and 6 months after the treatment was 8 and 2%, respectively (Table 3).

**Table 3:** Risk of recurrence/death per 1 U/ml CA-125 3 and 6 months after the treatment

Risk of	Time after treatment	HR	95%CI	P
Recurrence	3 months	1.083	1.05–1.12	< 0.0001
	6 months	1.018	1.01–1.03	< 0.0001
Death	3 months	1.08	1.03–1.13	0.0007
	6 months	1.02	1.01–1.03	0.0001

### CA-125 changes and risk of recurrence for selected groups of patients

Subgroups of patients were distinguished in terms of clinical and pathological factors (Table 4). Increase in CA-125 concentration by ≤5 U / ml did not affect the risk of relapse in any of the subgroups. In patients with FIGO stage 1 and 2 and those undergoing neoadjuvant chemotherapy, an increase in CA-125 > 5 U/ml 3 and 6 months after therapy may increase the risk of recurrence, but it was not statistically significant (*p* = 0.085, *p* = 0.09, *p* = 0.11, respectively). The highest risk of recurrence was observed in patients with the increase of CA-125 concentration > 5 U/ml receiving bevacizumab in maintenance therapy 6 months after the platinium-based chemotherapy.

**Table 4:** Risk of ovarian cancer recurrence and CA-125 serum concentration changes 3 and 6 months after therapy in subgroups of patients. NACT-Neoadjuvant chemotherapy

Subgroups of patients	Risk of ovarian cancer recurrence - HR (p)					
	CA-125 changes 3 months after therapy [U/ml]			CA-125 changes 6 months after therapy [U/ml]		
	< 0	0–5	> 5	< 0	0–5	> 5
**FIGO 1&2 (*n*** **= 52)**	1.00	0.97 (*p* = 0.97)	6.95 (*p* = 0.085)	1.00	1.57 (*p* = 0.516)	4.71 (*p* = 0.09)
**FIGO 3&4 (*n*** **= 116)**	1.00	1.42 (*p* = 0.145)	6.65 (*p* < 0.0001)	1.00	1.51 (*p* = 0.11)	6.33 (*p* < 0.0001)
**Grade 2&3 (*n*** **= 152)**	1.00	1.28 (*p* = 0.2885)	7.07 (*p* < 0.0001)	1.00	1.28 (*p* = 0.3388)	4.99 (*p* < 0.0001)
**Serous (*n*** **= 124)**	1.00	1.34 (*p* = 0.2247)	8.53 (*p* < 0.0001)	1.00	1.32 (*p* < 0.2955)	5.17 (*p* < 0.0001)
**NACT** **(*n*** **= 26)**	1.00	1.71 (*p* = 0.34)	4975 (*p* = 0.99)	1.00	1.22 (*p* = 0.698)	2.83 (*p* = 0.11)
**Bevacizumab (*n*** **= 32)**	1.00	1.62 (*p* = 0.29)	–	1.00	1.48 (*p* = 0.383)	10.1 (*p* < 0.008)
**Optimal cytoreduction (< 1 cm) (*n*** **= 145)**	1.00	1.34 (*p* = 0.4)	6.93 (*p* < 0.014)	1.00	1.64 (*p* = 0.179)	7.24 (*p* < 0.0001)

### CA-125 changes and risk of death

**Fig. 4:**
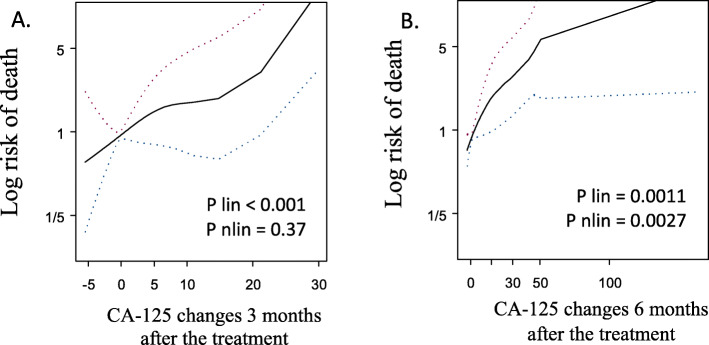
Risk of death according to CA-125 changes 3 (**a**) and 6 (**b**) months after the treatment. Both splins present increased risk of death along with increased change of CA-125. Linear effect was statistically significant 3 and 6 months after the treatment

**Fig. 5:**
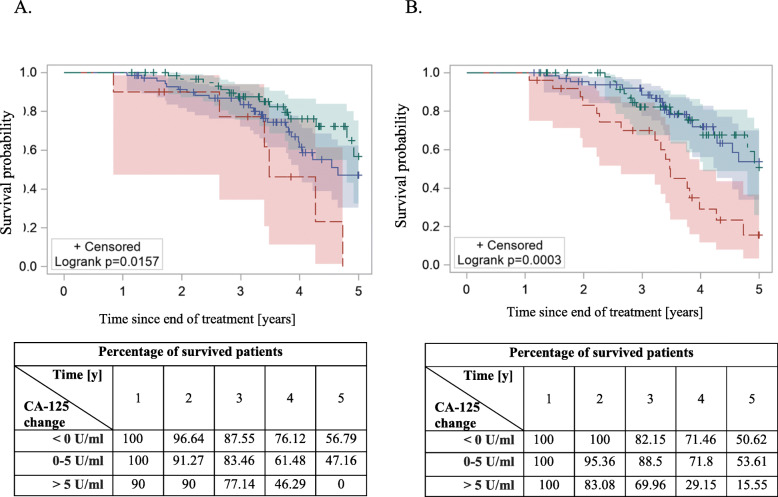
The 5-year overall survival Kaplan-Meier curves among patients with different CA-125 changes 3 (**a**) and 6 (**b**) months after end of the treatment. Green: < 0 U/ml; blue: 0–5 U/ml; red > 5 U/ml. Statistically significant difference in PFS was achieved with log-rank test

Patients with increase in CA-125 serum concentration > 5 U/ml had higher risk of death compared to those, whose concentration did not increase. It was shown that the risk of death 3 and 6 months after end of the treatment was HR = 3.83 (95% CI: 1.465–10.002, *p* < 0.006) and HR = 3.11 (95%CI: 1.528–6.308, *p* < 0.0017), respectively (Table 5, Figs. 4 and 5).

**Table 5:** Risk of death and CA-125 changes after the treatment

Variable		HR	95%CI	P
**Change of CA-125 concentration** **3 months after the treatment (U/ml)**	< 0	1.00	–	–
	0–5	1.62	0.84–3.11	0.15
	> 5	3.83	1.47–10.00	0.0062
**Change of CA-125 concentration** **6 months after the treatment (U/ml)**	< 0	1.00	–	–
	0–5	0.972	0.483–1.956	0.937
	> 5	3.1	1.53–6.31	0.0017

### CA-125 changes and risk of death for selected groups of patients

Risk of death was not corelated with increase in CA-125 concentration by ≤5 U / ml (Table 6). In patients with FIGO stage 3 and 4, high grade tumors (G2, G3) and serous histology an increase in CA-125 > 5 U/ml 3 and 6 months after treatment increased the risk of death. Patients who received bevacizumab consolidation therapy had the highest risk of death among analysed subgroups - increase of CA-125 concentration > 5 U/ml 6 months after chemotherapy was related to HR = 42.16 (*p* = 0.0025).

**Table 6:** Risk of ovarian cancer death and CA-125 serum concentration changes 3 and 6 months after therapy in subgroups of patients. NACT-Neoadjuvant chemotherapy

Subgroups of patients	Risk of ovarian cancer death - HR (p)					
	CA-125 changes 3 months after therapy [U/ml]			CA-125 changes 6 months after therapy [U/ml]		
	< 0	0–5	> 5	< 0	0–5	> 5
**FIGO 1&2 (*n*** **= 52)**	1.00	2.76 (*p* = 0.2065)	5.65 (*p* = 0.1636)	1.00	1.76 (*p* = 0.4893)	3.58 (*p* = 0.2080)
**FIGO 3&4 (*n*** **= 116)**	1.00	1.45 (*p* = 0.3157)	3.9 (*p* = 0.0121)	1.00	0.80 (*p* = 0.5824)	3.11 (*p* = 0.0037)
**Grade 2&3 (*n*** **= 152)**	1.00	1.90 (*p* = 0.0617)	3.91 (*p* = 0.006)	1.00	0.94 (*p* = 0.8647)	3.24 (*p* = 0.0015)
**Serous (*n*** **= 124)**	1.00	1.59 (*p* = 0.191)	4.4 (*p* = 0.0055)	1.00	0.91 (*p* = 0.8035)	2.79 (*p* = 0.0092)
**NACT** **(*n*** **= 26)**	1.00	5.14 (*p* = 0.1322)	7951 (*p* = 0.9994)	1.00	1.07 (*p* = 0.9291)	2.14 (*p* = 0.3347)
**Bevacizumab (*n*** **= 32)**	1.00	3.88 (*p* = 0.0915)	–	1.00	0.81 (*p* = 0.7677)	42.16 (*p* = 0.0025)
**Optimal cytoreduction (< 1 cm) (*n*** **= 145)**	1.00	2.21 (*p* = 0.128)	4.05 (*p* = 0.0958)	1.00	1.09 (*p* = 0.8648)	3.15 (*p* = 0.0405)

### Multivariate analyses for CA-125 changes

In multivariate analyses CA-125 changes > 5 U/ml 3 months after treatment significantly increased risk of recurrence HR = 85.19 (95% CI: 17.30–419.59; Table 7) and risk of death HR = 6.56 (95% CI: 1.37–31.39; Table 7). Increase of CA-125 by 0–5 U/ml was related to higher risk of recurrence HR = 2.78 (95%CI: 1.33–5.83), but not to risk of death HR = 2.63 (95%CI: 0.82–8.49). CA-125 change > 5 U/ml 6 months after the end of treatment was also related to the risk of recurrence HR = 19.00 (95%CI: 6.48–55.76; Table 8) and risk of death HR = 5.26 (95%CI: 1.12–24.85; Table 8).

**Table 7:** Final model for OC recurrence (A) and survival (B) for CA-125 changes 3 months after the treatment

Variable	Recurrence			Survival		
	HR	95%CI	***P***	HR	95%CI	***P***
CA-125 change < 0 U/ml	1.00	-	-	1.00	-	-
CA-125 change 0-5 U/ml	2.78	1.33-5.83	0.0068	2.63	0.82-8.49	0.1048
CA-125 change > 5 U/ml	85.19	17.30–419.59	0.0001	6.56	1.37-31.39	0.0185
Clear cell histology	6.01	1.59–22.66	0.0082	-	-	-
Endometrioid histology	0.05	0.01–0.42	0.0057	-	-	-
FIGO stage (I&II vs III&IV)	0.10	0.10	0.0004	-	-	-
High grade tumor	3.27	3.27	0.0242	3.22	0.98-10.55	0.0541

**Table 8:** Final model for OC recurrence (A) and survival (B) for CA-125 changes 6 months after the treatment

Variable	Recurrence			Survival		
	HR	95%CI	***P***	HR	95%CI	***P***
CA-125 change < 0 U/ml	1.00	-	-	1.00	-	-
CA-125 change 0-5 U/ml	3.68	1.45-9.30	0.0060	1.68	0.35-8.10	0.5199
CA-125 change > 5 U/ml	19.00	6.48-55.76	0.0001	5.26	1.12-24.85	0.0360
Clear cell histology	36.15	7.40-176.66	0.0001	16.62	1.21-228.85	0.0356
FIGO stage (I&II vs III&IV)	0.14	0.05-0.41	0.0003	-	-	-

## Discussion

CA-125 is a tumor marker of ovarian cancer that plays a significant role in making clinical decisions. Many studies assessed the importance of a single CA-125 measurement [7–13], while number of studies that analyzed the multiple evaluation of this marker is limited. Conducting such studies is challenging, as they long term follow up, large number of variables and due to difficulties in interpretation of the data. A further effort is related to the need of use of complex mathematical and statistical models. On the other hand, multiple measurements of tumor marker concentration yield additional information that cannot be obtained in a single assay. Such model can be used in screening, assessment of treatment effectiveness, prediction of relapse and overall survival.

Wilder et al. found association between increasing CA-125 concentration in the normal range and the risk of ovarian cancer recurrence [14]. Increasing CA-125 concentration in three consecutive samples by > 3% was consider as significant for ovarian cancer recurrence. The average time between the third increasing CA-125 value and relapse detection (clinically or radiologically) was 189 days (84–518 days). However, that study had some limitations. First, there was a small number of analyzed cases (*n* = 11) and second, the study group did not include patients without recurrence. The lack of opportunity to compare fluctuations in CA-125 concentration in the group of women with and without recurrence of the disease influenced the interpretation of the obtained results.

Santillan et al. compared changes in CA-125 concentration within the normal range.

within 22 patients with recurrence of ovarian cancer and 17 patients with disease remission [15]. It was shown that an increase in the concentration of 5 U/ml and 10 U/ml were significantly associated with the recurrence of the disease. The relative risk of recurrence in these patients was 8.4 (95% CI: 2.2–32.6, *p* = 0.002) and 71.2 (95% CI: 4.8 - > 999.9, *p* = 0.002), respectively. Moreover, it was noted that increase in CA-125 concentration by 100% from nadir is also strongly associated with the risk of recurrence (OR = 23.7, 95% CI: 2.9–195.2, *p* = 0.003). Our results confirm that the increase in CA-125 concentration by > 5 U/ml is significantly associated with the risk of relapse and also with risk of death. However, we analyzed CA125 changes 3 and 6 months after the treatment due to the significantly decreasing number of patients with CA-125 within normal range. The percentage of patients with CA125 concentrations within reference values 3, 6 and 9 months after end of the treatment were 83.3, 76.2 and 55.3%, respectively. To preserve representative study group we decided to limit the analysis to two groups: 3 and 6 months after the treatment. As a result, it was possible to identify a group of patients with a high risk of relapse. Moreover, due to the unspecific nature of the CA-125, the longer observation period, the greater risk of incorrect interpretation of the marker fluctuations.

We showed that increasing CA-125 by > 5 U/ml is associated with risk of recurrence, but hazard ratios in 3 and 6 months were different (HR = 7.6 and HR = 5.29, respectively). This indicated that the risk of recurrence is not constant for a specific CA125 value, but it changes over time. Assessment of changes in CA-125 concentration without taking into account the time from the end of treatment may lead to wrong conclusions.

Wang et al. used the ROC curve to determine the CA-125 serum level characteristics for recurrence in patients with complete remission [16]. They used nadir as a reference level and 1.68 x nadir was determined as significant for recurrence with 82.9% sensitivity and 85.6% specificity.

Liu et al. defined early ovarian cancer progression (EPD - Early Progression Disease) [17]. According to this definition, early relapse was diagnosed if: 1) CA-125 > 20 U/ml (for patients with CA-125 nadir < 10/ml), 2) 2 x nadir CA-125 (for patients with CA-125 nadir > 10/ml). These criteria were applied retrospectively on 288 patients. OC recurrence was diagnosed in 204 patients, of which 56% met EPD. There were 9 false positive cases in the group of women without relapse.

Levy et al. compared criteria detecting early OC recurrence based on different increase in CA-125 concentration within the normal range [18]: 1) increase of CA-125 concentration by 5 U/ml above nadir, 2) EPD criteria described by Liu et al., 3) increase of CA-125 concentration > 20 U/ml. In patients with OC recurrence, the criteria were met in 72.4, 53.4 and 63.8%, respectively. Although 24 patients were in remission, false positive results were obtained in 50, 8.3, 20.8%, respectively. Negative predictive value was 42.8, 44.9 and 47.5%, respectively. Authors concluded that use of EPD criteria in clinical practice, despite high specificity (91.7%) and positive predictive value (93.9%), is limited due to low sensitivity (53.3%) and negative predictive value (44.9%).

In the presented study two intervals were distinguished: 1) between the end of treatment and the 3rd month of observation and 2) between the end of treatment and the 6th month of observation. Risk of recurrence and risk of death were significantly higher in patients with increasing CA-125 concentration by > 5 U/ml at both time intervals. Using the same cut-off values for 3 and 6 months follow-up it was possible to compare risks over time. In the previous studies, it was assumed that the recurrence risk depends on the concentration of CA-125 (or its change) within reference range. Various criteria of CA-125 concentration changes within the normal range were evaluated, showing a variable prognostic value. In total, patients with recurrences in the early post-treatment period and patients with long-term remission were analyzed. Our results indicate that an increase in > 5 U / ml in the third month after the end of treatment is associated with more than a 7-fold recurrence risk and almost a 4-fold risk of death (Table 2, Table 5). The same change in concentration, but after 6 months follow up is associated with a 5-fold risk of recurrence and 3-fold risk of death. As a result, it was shown that the recurrence risk is not constant for a given change in CA-125 concentration.

Previously published studies have shown that increasing CA-125 levels within the normal range may be an early signal of OC recurrence. However, they focused on establishing a cut-off level or increasing concentration of CA-125 below 35 U/ml. In these studies, it was assumed that the recurrence risk depends on the concentration of CA-125 (or its change) within reference range. Unfortunately, false positive results (8.3–50%) and negative predictive values (42.8–47.5%) prevent using these criteria in clinical practice. This may be caused by excluding the time since the end of the treatment and the assumption that the recurrence risk depends on the concentration of CA-125 (or its change). Our results showed that the recurrence risk indicated by the increase in CA-125 concentration is variable over time. The same increase in CA-125 concentration in several points of time is associated with different risk of relapse. Therefore, interpretation of the same CA-125 changes in the short- and long-term observation should be different.

Our results may indicate that the longer period of time since the end of treatment, the CA-125 changes within the normal range may be a weaker predictor of early relapse, especially in patients with comorbidities that cause serum CA-125 increase. In the early post-treatment period (3 to 6 months after therapy), CA-125 monitoring can stratify patients to the risk of relapse. Patients classified as high-risk OC relapse should be closely monitored in oncological centers. While follow-up in patients with low risk of recurrence could be conducted by gynecology-oncologists or oncologist in regional hospitals. The frequency of follow-up visits could be different among high and low risk patients as well.

Nowadays CA-125 monitoring during follow-up is not obligatory and relapse is diagnosed mainly clinically or radiologically. Patients are classified as platinum-resistant/(partially) sensitive according to time since the end of the treatment until OC recurrence. Based on this classification (not only) second-line chemotherapy is made. While detection of OC recurrence is made more than 12 months after the end of treatment, increase of CA-125 concentration within the normal range in the first months after treatment may indicate resistance to platinum-based chemotherapy.

Moreover, there was a statistically significant risk of OC recurrence per 1 U/ml 3 and 6 months after the end of treatment - 8.3 and 1.8% respectively. It confirms that the risk of relapse is not constant over the time and depends on the length of the observation. Conversion per unit allows to compare the risk of recurrence between patients. The difference in CA-125 concentration 3 months after the end of the treatment of 4 U/ml means that the difference in the risk of recurrence is 33.2% (4 × 8.3%).

According to clinicopathological factors subgroups of patients were established (Table 4). In none of them, the increase in CA-125 concentration by 0–5 U/ml was not associated with an increased risk of relapse. Although anti-angiogenic therapy may affect CA-125 concentration and impair its significance [19], it was shown that increase in CA-125 by > 5 U/ml in patients, who received bevacizumab was associated with the higher risk of recurrence (HR = 10.1, *p* < 0.008). FIGO I/II patients (*n* = 52) and FIGO III/IV, who started treatment with NACT (*n* = 26) had increased risk of recurrence, but not statistically significant (*p* = 0.085, *p* = 0.09, *p* = 0.11). The trend was the same, but more research is needed on these two subgroups.

Our study had several limitations. Retrospective design of the study made it prone to selection bias. Presented results refer to patients with ovarian cancer, who achieved complete radiological, clinical and biochemical remission. These results are not representative for patients with residual disease despite the absence of clinical symptoms and normalization of CA-125. The study group predominantly consisted of patients with serous, high grade ovarian cancer. It is not therefore certain whether these results could be translated to other histopathological types.

## Conclusions

1. Increase of CA-125 serum concentration by > 5 U/ml within the normal range 3 and 6 months after the end of treatment in patients who achieved complete remission is independent poor prognostic factor and significantly raises the risk of OC recurrence and risk of death.
2. The risk of OC recurrence related to serum CA-125 concentration changes over time. The longer the time since the end of treatment, the lower the risk of recurrence/death related to fluctuations of CA-125 within normal range.

## Methods

Inclusion criteria included: diagnosis of epithelial ovarian cancer, surgery treatment followed by chemotherapy, complete remission (clinical: no signs and symptoms, radiological: complete response according to RECIST 1.1 and biochemical: CA-125 < 35 U/ml), regular follow-up (every 3 +/− 1 months for the first 2 years after the end of treatment, every 6 +/− 1 months for the next 3 years). Serum CA-125 concentration was assessed on each appointment. The minimum follow-up period was 12 months. The end points of the study were disease relapse and patient’s death. Recurrence of the disease was diagnosed on the basis of clinical symptoms and radiological criteria whichever occurred first. In some cases, biopsy or surgery was done to confirm recurrence in microscopic examination.

### CA-125 measurements

CA-125 assay was performed up to 14 days before appointment. Laboratory tests were performed with electrochemiluminescence immunoassay using the cobas e 601 analyzer (Roche Diagnostics) according to manufacturer’s protocol [20]. Based on CA-125 changes in concentration after end of the treatment, 3 groups of patients were established:

- CA-125 concentration has not increased
- CA-125 concentration increased ≤5 U/ml
- CA-125 concentration increased > 5 U/ml

Bioethical Committee of Medical University of Warsaw approved the study.

### Statistical analysis

The descriptive part of the statistical analysis consisted of presenting the distributions (expressed as a percentage) for qualitative variables and the means with standard deviations or medians for quantitative variables. The main statistical analysis included Progression Free Survival (PFS) and Overall Survival (OS) in the 5-year observation period based on the Kaplan-Meier estimaton and plotting the relevant curves. Relationships between quantitative variables and the risk of recurrence/death were conducted using generalized additive models applied to the Cox proportional hazards method to determine curves illustrating linear and non-linear dependence. Statistically significant nonlinear effect was the basis for determination subgroups of patients for whom the Kaplan-Meier curves were drawn. The percentage of relapses/deaths in the 5-year follow-up period was compared between the subgroups of patients using the log rank test. The calculations used the FREQ, UNIVARIATE, NPAR1WAY and LIFETEST procedures of the SAS system.

The methodological aspects are based on the textbook van Belle et al. [21], while the technical and methodological aspects of the work were discussed in the documentation: SAS / STAT® 9.4 / 14.4, User’s Guide, SAS Institute Inc., Cary, NC, USA, 2017.

Apart of CA-125 changes 3 and 6 months after the treatment the following concomitant variables were considered in multivariate analyses: primary surgery, NACT, degree of cytoreduction (R0 vs R < 1 cm vs R > 1 cm), histology, staging, grading, number of total chemotherapy cycles, maintenance therapy with bevacizumab, age, duration of treatment (from diagnosis to the end of the chemotherapy), CA-125 nadir level. All of these mentioned variables were considered as putative risk factors for cancer recurrence or death in multiple analysis. Due to nonlinear significant relation between CA-125 changes and risks of cancer recurrence and death this parameter was used in analysis both as continuous (per 1 U/ml) and categorical variable (< 0 U/ml, 0–5 U/ml and > 5 U/ml). Forward selection was performed to find final models.

## Data Availability

The datasets used and/or analysed during the current study are available from the corresponding author on reasonable request.
